# Identification of a novel *de novo* ANK1 R1426* nonsense mutation in a Chinese family with hereditary spherocytosis by NGS

**DOI:** 10.18632/oncotarget.18243

**Published:** 2017-05-27

**Authors:** Xiong Wang, Bin Yi, Ketao Mu, Na Shen, Yaowu Zhu, Qun Hu, Yanjun Lu

**Affiliations:** ^1^ Department of Laboratory Medicine, Tongji Hospital, Tongji Medical College, Huazhong University of Science and Technology, Wuhan 430030, China; ^2^ Department of Pediatric Surgery, Tongji Hospital, Tongji Medical College, Huazhong University of Science and Technology, Wuhan 430030, China; ^3^ Department of Radiology, Tongji Hospital, Tongji Medical College, Huazhong University of Science and Technology, Wuhan 430030, China; ^4^ Department of Pediatrics, Tongji Hospital, Tongji Medical College, Huazhong University of Science and Technology, Wuhan 430030, China

**Keywords:** hereditary spherocytosis, splenectomy, anemia, ankyrin, mutation

## Abstract

Hereditary spherocytosis (HS) is an inherited heterogeneous hemolytic anemia, characterized by the presence of spherical-shaped erythrocytes on the peripheral blood smear, and the clinical manifestation ranges from asymptomatic to severely anemic, and transfusion-dependent patients. Mutations in at least five genes (ANK1, EPB42, SLC4A1, SPTA1, and SPTB) have been identified so far, and mutations of ANK1 gene are responsible for the majority of all HS cases. In this study, targeted next generation sequencing (NGS) was applied to identify a novel *de novo* ANK1 c.4276C>T (p.R1426*) nonsense mutation in a Chinese family with a patient of HS who was diagnosed clinically with only 10% spherical-shaped erythrocytes in the peripheral blood and received splenectomy. Sanger sequencing further confirmed that only the patient carried heterozygous ANK1 c.4276C>T nonsense mutation, while none of his parents or his young brother carried this mutation. Moreover, consistent with the genetic findings, the anemia was ameliorated after splenectomy. RBCs increased from 2.74 × 1012/L pre-surgery to 4.76 × 1012/L one month post-surgery, and hemoglobin increased from 66g/L to 126g/L respectively. This is the first report of ANK1 c.4276C>T (p.R1426*) heterozygous nonsense mutation responsible for HS. Our results also demonstrate that targeted NGS may provide a powerful approach for rapid genetic test of HS.

## INTRODUCTION

Hereditary spherocytosis (HS) is the most frequent form of inherited hemolytic anemia characterized by the presence of spherical-shaped erythrocytes on the peripheral blood smear, hemolysis, splenomegaly, jaundice, and gallstones [1]. The clinical severity of HS varied widely, and its prevalence is 1 in 2,000 in Northern European population, and the prevalence in China is about 1 in 100,000 people [2]. To date, mutations in at least five genes (*ANK1*, *EPB42*, *SLC4A1*, *SPTA1*, and *SPTB*), encoding ankyrin, protein 4.2, band 3 protein, α-spectrin, and β-spectrin respectively, are associated with HS.

The majority of HS cases is associated with autosomal dominant (AD) pattern, although autosomal recessive (AR) inheritance has also been described. Mutations in *ANK1* (mainly nonsense and frame shift mutations) are responsible for about 75% of cases of HS in humans, followed by mutations in *SLC4A1* and *SPTB* genes [3, 4]. Ankyrin typically consists of three structural domains: a multiple ankyrin repeats N-terminal domain, a spectrin-binding center region, and a regulatory C-terminal domain, which is the least conserved [5, 6]. Erythroid ankyrin is a major protein in RBCs involved in the linkage of transmembrane proteins and the cell membrane skeleton via spectrin, band 4.2 protein, band 3 protein, and ankyrin deficiency results in reduced incorporation of spectrin [7]. Up to now, 59 distinct *ANK1* mutations have been reported in human HS in HGMD database (www.hgmd.cf.ac.uk), including deletion, frameshift, nonsense, or missense mutations.

Recent advances in next generation sequencing (NGS) approach provide rapid detection of genetic mutations, especially for large panel including a variety of candidate genes, leading the field of genetic testing away from Sanger sequencing [8]. In this study, we analyzed the disease-causing mutations in a Chinese family with a patient of HS using targeted NGS to identify the mutational characteristics in *ANK1*, *EPB42*, *SLC4A1*, *SPTA1*, and *SPTB* genes, followed by Sanger sequencing confirmation.

In this study, a novel *de novo ANK1* c.4276C>T (p.R1426*) heterozygous nonsense mutation in the patient characterized with only 10% spherical-shaped erythrocytes in the peripheral blood, anemia, and splenomegaly, while none of his parents or his young brother carried this mutation. Moreover, consistent with the genetic findings, the anemia was ameliorated after splenectomy. This study also demonstrates that targeted NGS is an effective method for identifying causal mutations for HS diagnosis.

## RESULTS

### Clinical features of a chinese family with HS

**Figure 1:**
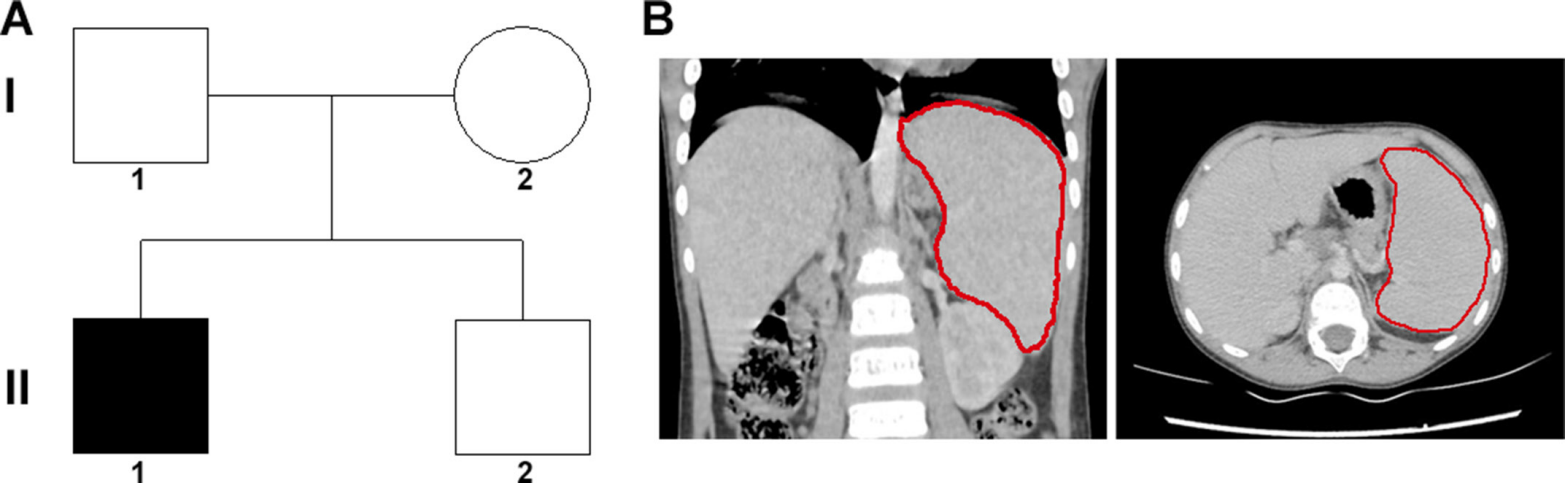
Family tree and clinical characteristics of the patient (**A**) the family tree of a Chinese family with patient of HS. A filled square was patient, and unfilled square or circle denoted unaffected male or female respectively. (**B**) the re-constructed CT scanning image, and the red line outlined the spleen.

An 8 years old male patient with anemia, jaundice, and splenomegaly, received splenectomy due to severe anemia although only 10% spherical-shaped erythrocytes on the peripheral blood smear. The family tree was drawn (Figure 1A), and the splenomegaly identified by CT scanning was shown in Figure 1B. The changes of RBC and Hemoglobin of the patient with HS pre/post-surgery was recorded in Table 1, and the anemia was ameliorated after splenectomy.

**Table 1:** RBC and Hemoglobin changes of the patient with HS

Test	3 Months Pre-operation	1 DayPre-operation	1 Week Post-operation	1 Month Post-operation
RBC (×10^12^/L)	2.52	2.74	4.52	4.76
Hemoglobin (g/L)	58.0	66.0	118.0	126.0

### NGS output and coverage

**Figure 2:**
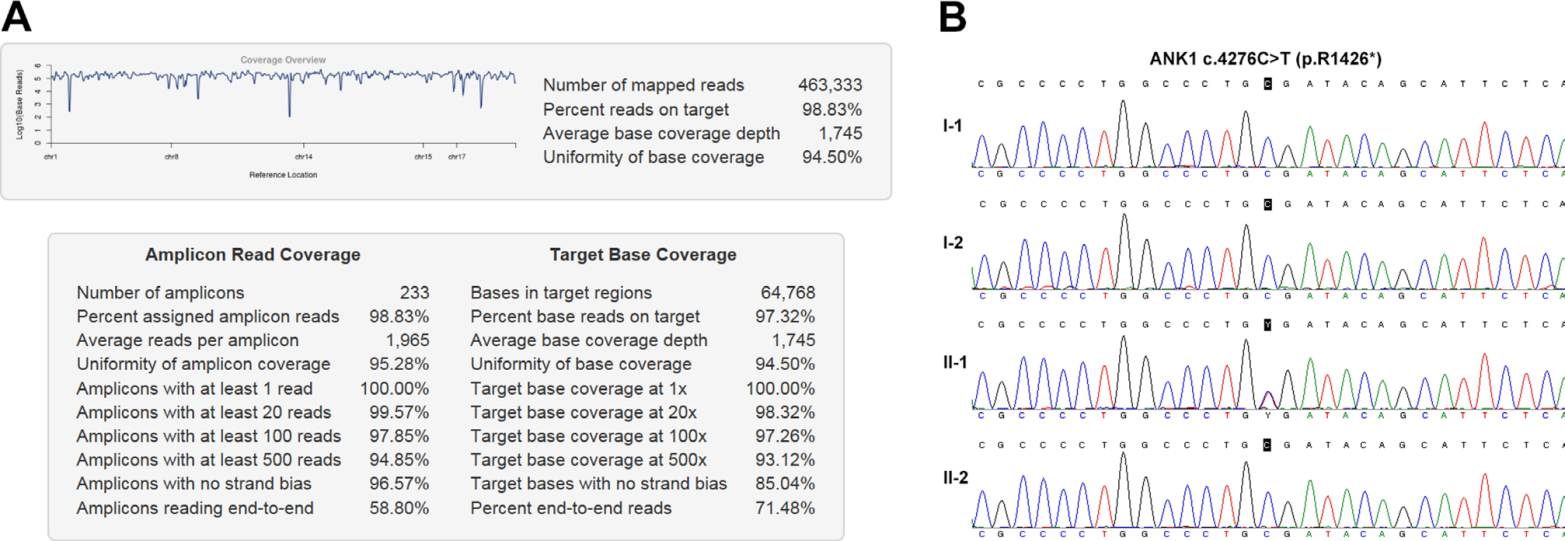
NGS output and mutation analysis (**A**) The coverage summary of targeted NGS for HS genetic diagnosis. (**B**) ANK1 c.4276C>T (p.R1426*) mutation analysis.

The sequencing of coding exons and adjcant intronic regions of 5 HS-associated genes on the Ion torrent PGM achieved an average output of 463,333 mapped reads and 98.83% on target. In summary, 100% of all target amplicons was covered at least once, 99.57% amplicons was covered at least 20 times, 97.85% amplicons was covered at least 100 times. The mean uniformity of base coverage is 94.50% in this panel. The average read depth was 1745 folds (Figure 2A).

### Mutation detection and sanger sequencing validation

NGS identified a total of 40 variants in the patient subjected to a process to discover pathogenic mutations (Supplementary Table 1). After applying filtration process and annotation, a heterozygous *ANK1* c.4276C>T (p.R1426*) nonsense mutation was identified which could be implicated in the patient’s phenotype.

Sanger sequencing of this loci was performed on all immediate family members. The result showed that only the patient carried this mutation, while none of his parents or his young brother held this mutation (Figure 2B). Paternity testing by haplotype analysis confirmed that these were the biological parents of the patient with HS (Table 2). These data indicate a *de novo* mutation.

**Table 2:** Four SNPs selected for paternity testing

SNP	I-1 (Father)	I-2 (Mother)	II-1 (Proband)	II-2 (Young brother)
rs2278621	CT	CC	CT	CC
rs504574	CG	CC	CG	CG
rs7826127	CT	TT	CT	TT
rs1872877	AG	AG	GG	GG

Moreover, *ANK1* c.4276C>T mutation (hg19: chr8:41546059G>A) was not found in RBCmembrane, 1000G, ExAC, HGMD, or EVS database.

### Genetic and phenotype association analysis

Genetic tests showed a *de novo ANK1* c.4276C>T (p.R1426*) nonsense mutation in this family, and only the patient carried this mutation. Hemolysis, splenomegaly, and jaundice were observed only in the patient. Moreover, consistent with the genetic findings, the anemia was ameliorated after splenectomy, a reserved surgery for the treatment of HS patients with severe symptoms. RBCs increased from 2.74 × 10^12^/L pre-surgery to 4.76 × 10^12^/L one month post-surgery, and hemoglobin increased from 66 g/L to 126 g/L respectively (Table 3).

**Table 3:** Primer sequences used in this study

Primer name	Forward	Reverse	Target
ANK1 CD4	GTCATAGAGAACAGGCTGGATC	GGCTTAGGTCCAAAACAATTC	rs1872877
ANK1 CD9	CTGGTGCTTGAGGAAACG	AAGTTTGCCGACCCTGAT	rs7826127
ANK1 CD27	CACCGAGACCTCAGACAACA	TCTCAGGGCTATGGACACC	rs504574
ANK1 CD34	GTCTGGCCATGCCTGTAAAG	AGGGTCCACAGCGTGAATG	rs2278621
ANK1 CD35	CTTCACTCCCACTCCTCCC	CTGCAAGATCAGGGGAAGAC	c.4276C>T

### Functional prediction of the mutation

Ankrin includes an 89kDa domain in N-terminal, a UPA domain in the center, and a 55kDa regulatory domain in C-terminal which is involved in regulating binding of β-spectrin and band 3 protein. *ANK1* c.4276C>T nonsense mutation produced a short protein (p.R1426*) located in the 55kDa regulatory domain in C-terminal and resulted in premature termination of ankyrin.

Two kinds of software were applied to predict the effect of this mutation. *ANK1* c.4276C>T nonsense mutation was predicted as ‘disease causing’ in MutationTaster, and ‘Pathogenic’ in InterVar with evidences of PVS1, PS2, and PM2 [9].

## DISCUSSION

*ANK1* mutation is the most common cause of HS, followed by mutations in *SPTB*, *SPTA1*, *SLC4A1*, and *EPB42* (∼20%, ∼5%, ∼15%, and ∼10% respectively) [10]. *ANK1* mutation is inherited in both AD and AR patterns in HS and the majority is *de novo* mutation [11]. To date, 7 missense mutations, 15 nonsense mutations, 7 splicing mutations, 3 regulatory mutations, 19 small deletions, 5 small insertions, 1 small indel, 1 gross deletion, 1 gross insertion/duplication have been identified among patients with HS in HGMD database. 7 of the 15 nonsense mutations, accounting for about half of all nonsense mutations, were located in the 55kDa regulatory domain in C-terminal of ankrin which modulates the affinities of the other domains and contains a death domain[6]. The regulatory domain modifies the association of ankyrin with the band 3 protein complex and the RBC cytoskeleton network [12]. Deficient ankyrin due to nonsense mutations in the regulatory region have been proposed as causes of HS [13]. Hughes MR et al. found that mice with truncation mutation of ankyrin lacking the spectrin-binding and regulatory C-terminal domains modeled severe hemolytic *in vivo* [14]. Some other proteins may also contribute to red cell skeleton, including the Rh complex, e.g. CD47 which interacts with protein 4.2 [15].

In our study, hemolysis and jaundice were found in the patient, and splenomegaly was identified by CT scanning. Genetic tests showed a *de novo ANK1* c.4276C>T (p.R1426*) nonsense mutation in the patient, while none of his patients or his young brother carried this mutation. *ANK1* c.4276C>T mutation was novel, and was not found in RBCmembrane, 1000G, ExAC, HGMD, or EVS database.This mutation was located in the regulatory domain in C-terminal, which might cause HS. Moreover, consistent with the genetic diagnosis, the anemia was ameliorated after splenectomy, further supporting the genotype-phenotype relationship. Our results expanded the mutation spectrum of *ANK1* in HS.

High-throughput NGS has promoted DNA sequencing by allowing the cost-effective and simultaneous rapid detection of multiple genes, especially in cases where traditional laboratory tests failed, or when the patients were extensively transfused [16]. Agarwal AM et al. have designed a panel including 28 genes which encode both RBC cytoskeletal proteins and enzymes to perform genetic test for hereditary haemolytic anemia [17]. Han JH et al. performed whole exome sequencing (WES) to identify casual mutation in a Korean family with HS patient [13]. We have successfully used a panel covering the complete coding region, splice site junctions, and UTR regions of the previously reported five HS related genes (OMIM#182900, #616649, #270970, #612653, and #612690) to determine the molecular basis of patients with HS on Ion torrent PGM platform. These studies demonstrate the clinical utility of WES or targeted NGS could provide rapid genetic diagnosis for patients with inherited haemolytic anemia.

In summary, a novel *de novo ANK1* c.4276C>T (p.R1426*) nonsense mutation was identified in a Chinese family affected by HS with only 10% spherical-shaped erythrocytes combined with jaundice, and splenomegaly. Moreover, consistent with the genetic findings, the anemia was ameliorated after splenectomy. This is the first report of *ANK1* c.4276C>T (p.R1426*) heterozygous nonsense mutation responsible for HS. Our results also demonstrate that NGS may provide a powerful approach for rapid genetic test of HS.

## MATERIALS AND METHODS

### Subjects

The male patient showed anemia and jaundice half a year after birth (hemoglobin: 95 g/L), was diagnosed with HS at 5 years old with 4.3% spherical-shaped erythrocytes on the peripheral blood smear and received blood transfusion for several time. At 8 years old, the genetic test upon the patient and his immediate family members was performed three months before a splenectomy due to anemia and splenomegaly with 10% spherical-shaped erythrocytes on the peripheral blood smear. The patient’s father was 40 years old, and his mother was 32 years old. One month after the splenectomy, his hemoglobin and RBCs levels improved to normal range dramatically. Written informed consent was obtained from all participants. This study was formally approved by the Ethics Committee of Tongji Hospital, Tongji Medical College, Huazhong University of Science and Technology. All procedures were performed in accordance with the approved guidelines.

### Library preparation and Ion-Torrent PGM sequencing

Ion Torrent adapter-ligated libraries were built using Ion Ampliseq^™^ Library Kit 2.0 (Thermo Fisher Scientific). Briefly, 10 ng genomic DNA from peripheral blood mononuclear cell (PBMC) quantitated by Qubit 2.0 (Invitrogen, Carlsbad, CA, USA) was used for multiplex PCR amplification in two primer-pools with 118 and 115 amplicons respectively. The PCR products of the two pools were mixed together, and ligated to barcodes and Ion Torrent adapters (Life Technologies), followed by purification with AMPure XP beads (Beckman Coulter, Brea, CA, USA) and quantification by Ion Library TaqMan™ Quantitation Kit. Barcoded libraries of different samples were pooled in equal amount and used as template for emulsion PCR using the PGM^™^ Hi-Q^™^ OT2 Kit on the Ion One Touch 2 Instrument, followed by enrichment of Ion Sphere Particles (ISPs) on the ES Instrument. Finally, 850 flows sequencing was done on the Ion 316^™^ Chip v2 using Ion PGM Hi-Q Sequencing Kit on the Ion Torrent Personal Genome Machine (PGM).

### Bioinformatic analysis

**Figure 3:**
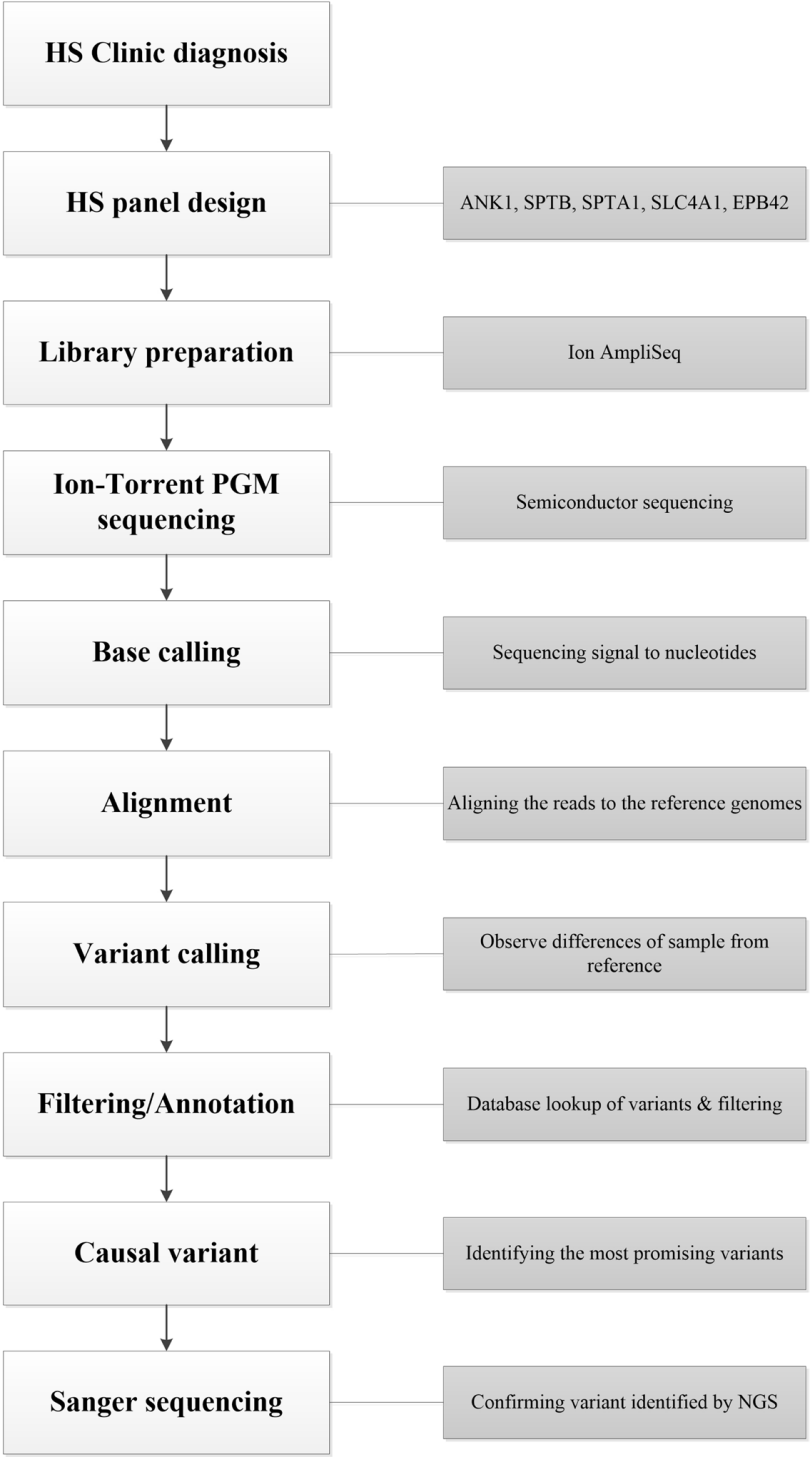
The workflow of this work

Raw sequence data were processed to trim adapter sequences, align to the hg19 human reference genome (GRCh37), analyze coverage and call variants on the Ion Torrent Server using the Torrent Suite software version 4.4.2 and the Variant Caller plugin, with the default parameters suggested by the manufacturer. Variant analysis was performed with the Ion Reporter 5.0 software to classify variants as single nucleotide variations (SNVs), multiple nucleotide variants (MNV), insertions or deletions (indels). All found variants and regions below 20 × coverage were visually verified with the Integrative Genomics Viewer (IGV) v2.3.8 (Broad Institute). Variants were annotated according to the nomenclature recommended by the Human Genome Variation Society (HGVS, http://www.hgvs.org) and their classification was attributed according to the Red Cell Membrane Disorder Mutations Database (RBCmembrane, http://research.nhgri.nih.gov/RBCmembrane), ClinVar https://www.ncbi.nlm.nih.gov/clinvar/, 1000G (http://asia.ensembl.org/index.html), HGMD (http://www.hgmd.cf.ac.uk/ac/index.php), ExAC (http://exac.broadinstitute.org/), EVS (http://evs.gs.washington.edu/EVS/), and InterVar (http://wintervar.wglab.org/) databases [9]. To predict possible impact of non-synonymous variants in exons, in-silico function was predicted by several software, including SIFT (http://sift.jcvi.org/www/SIFT_BLink_submit.html), MutationTaster (http://www.mutationtaster.org/), MutationAssessor (http://mutationassessor.org), Provean http://provean.jcvi.org/index.php and PolyPhen-2 (http://genetics.bwh.harvard.edu/pph2/) [18–21]. The whole workflow was summarized in Figure 3.

### Sanger sequencing

Sanger sequencing was performed as previously described [22]. Breifly, genomic DNA was extracted from PBMC using QIAamp DNA blood mini kit (QIAGEN, Hilden, Germany). Coding exons and splice junctions of ANK1, EPB42, SLC4A1, and SPTB genes (GenBank accession no. NM_001142446.1, NM_000119.2, NM_000342.3, NM_003126.2, and NM_001024858.2, respectively) were amplified for identified mutation, failed amplicons, or missed target region for amplicon design. Sanger sequencing of PCR products was performed bi-directionally on ABI 3500 Dx Genetic Analyzer (Applied Biosystems). Sequence was blasted on NCBI.

### Paternity testing and haplotype analysis

Four single nucleotide polymorphisms (SNPs; rs2278621, rs504574, rs7826127, and rs1872877) ranging from chr8: 41690142 to chr8: 41728053 were selected from the UCSC Genome Browser (http://genome.ucsc.edu/), and linkage-disequilibrium analysis was performed based on LD TAG SNP selection (TagSNP; http://snpinfo.niehs.nih.gov/snpinfo/snptag.php). All primer sequences were listed in Table 3.

## SUPPLEMENTARY MATERIALS TABLE





## References

[1] Perrotta S, Gallagher PG, Mohandas N (2008). Hereditary spherocytosis. Lancet.

[2] Wang C, Cui Y, Li Y, Liu X, Han J (2015). A systematic review of hereditary spherocytosis reported in Chinese biomedical journals from 1978 to 2013 and estimation of the prevalence of the disease using a disease model. Intractable Rare Dis Res.

[3] Agre P, Orringer EP, Bennett V (1982). Deficient red-cell spectrin in severe, recessively inherited spherocytosis. N Engl J Med.

[4] Gallagher PG (2005). Hematologically important mutations: ankyrin variants in hereditary spherocytosis. Blood Cells Mol Dis.

[5] Platt OS, Lux SE, Falcone JF (1993). A highly conserved region of human erythrocyte ankyrin contains the capacity to bind spectrin. J Biol Chem.

[6] Rubtsov AM, Lopina OD (2000). Ankyrins. FEBS Lett.

[7] Delaunay J (2007). The molecular basis of hereditary red cell membrane disorders. Blood Rev.

[8] Koboldt DC, Steinberg KM, Larson DE, Wilson RK, Mardis ER (2013). The next-generation sequencing revolution and its impact on genomics. Cell.

[9] Li Q, Wang K (2017). InterVar: Clinical interpretation of genetic variants by the 2015 ACMG-AMP guidelines. Am J Hum Genet.

[10] An X, Mohandas N (2008). Disorders of red cell membrane. Br J Haematol.

[11] Miraglia del Giudice E, Nobili B, Francese M, D’Urso L, Iolascon A, Eber S, Perrotta S (2001). Clinical and molecular evaluation of non-dominant hereditary spherocytosis. Br J Haematol.

[12] Hall TG, Bennett V (1987). Regulatory domains of erythrocyte ankyrin. J Biol Chem.

[13] Han JH, Kim S, Jang H, Kim SW, Lee MG, Koh H, Lee JH (2015). Identification of a novel p.Q1772X ANK1 mutation in a Korean family with hereditary spherocytosis. PLoS One.

[14] Hughes MR, Anderson N, Maltby S, Wong J, Berberovic Z, Birkenmeier CS, Haddon DJ, Garcha K, Flenniken A, Osborne LR, Adamson SL, Rossant J, Peters LL (2011). A novel ENU-generated truncation mutation lacking the spectrin-binding and C-terminal regulatory domains of Ank1 models severe hemolytic hereditary spherocytosis. Exp Hematol.

[15] Mouro-Chanteloup I, Delaunay J, Gane P, Nicolas V, Johansen M, Brown EJ, Peters LL, Van Kim CL, Cartron JP, Colin Y (2003). Evidence that the red cell skeleton protein 4.2 interacts with the Rh membrane complex member CD47. Blood.

[16] Lu JT, Campeau PM, Lee BH (2014). Genotype-phenotype correlation—promiscuity in the era of next-generation sequencing. N Engl J Med.

[17] Agarwal AM, Nussenzveig RH, Reading NS, Patel JL, Sangle N, Salama ME, Prchal JT, Perkins SL, Yaish HM, Christensen RD (2016). Clinical utility of next-generation sequencing in the diagnosis of hereditary haemolytic anaemias. Br J Haematol.

[18] Kumar P, Henikoff S, Ng PC (2009). Predicting the effects of coding non-synonymous variants on protein function using the SIFT algorithm. Nat Protoc.

[19] Schwarz JM, Cooper DN, Schuelke M, Seelow D (2014). MutationTaster2: mutation prediction for the deep-sequencing age. Nat Methods.

[20] Choi Y, Chan AP (2015). PROVEAN web server: a tool to predict the functional effect of amino acid substitutions and indels. Bioinformatics.

[21] Adzhubei I, Jordan DM, Sunyaev SR (2013). UNIT 7.20 Predicting Functional Effect of Human Missense Mutations Using PolyPhen-2. Curr Protoc Hum Genet.

[22] Wang X, Zhu Y, Shen N, Peng J, Wang C, Liu H, Lu Y (2016). Mutation analysis of a Chinese family with oculocutaneous albinism. Oncotarget.

