# Supplementary material for: Identification of a novel de novo ANK1 R1426* nonsense mutation in a Chinese family with hereditary spherocytosis by NGS
Source: Oncotarget. 2017 May 27;8(57):96791–7. doi: 10.18632/oncotarget.18243 (PMC5722523; doi:10.18632/oncotarget.18243)
Supplement: Supplementary file 1 [file oncotarget-08-96791-s001.pdf]

Identification of a novel *de novo* ANK1 R1426* nonsense mutation in a Chinese family with hereditary spherocytosis by NGS

Supplementary Materials

Supplementary Table 1: Variants identified by NGS. See Supplementary_Table_1
